# The causal effects between selenium levels and the brain cortical structure: A two‐sample Mendelian randomization study

**DOI:** 10.1002/brb3.3296

**Published:** 2023-10-30

**Authors:** Xiaowei Zhang, Yuqing Zhong, Kejun He

**Affiliations:** ^1^ Department of Neurosurgery The First Affiliated Hospital of Sun Yat‐sen University Guangzhou China; ^2^ The First Affiliated Hospital of Sun Yat‐sen University Guangzhou China

**Keywords:** Cortical structure, Mendelian randomization analysis, Selenium

## Abstract

Extensive research has demonstrated the critical role of selenium (Se) and selenoproteins in brain function and cognition. However, the impact of Se on brain cortical structure remains enigmatic. Therefore, this study used Mendelian randomization (MR) analysis to investigate the causal effect between Se levels and brain cortical structure.

**Methods:**

This study utilizes 11 genetic variants associated with Se level variations, extracted from a large‐scale genome‐wide association study (GWAS) encompassed circulating Se levels (*n* = 5477) and toenail Se levels (*n* = 4162) in the European population. Outcome data were sourced from the summary statistics of the ENIGMA Consortium, comprising GWAS data from 51,666 individuals. The variables include cortical surface area (SA), thickness (TH) at the global level, and 34 functional cortical regions evaluated by magnetic resonance imaging. The inverse‐variance‐weighted method was used as the primary estimate. Additionally, sensitivity analyses were conducted to detect potential violations of assumptions underlying MR.

**Results:**

At the global level, Se levels were not correlated with SA but showed a significant negative correlation with TH (*β* = −0.00485 mm, SE = 0.00192, *p* = .0115). Heterogeneity was observed across different brain regions, with positive correlations found between Se levels and the TH of the parahippocampal gyrus, superior frontal gyrus, and frontal pole, whereas negative correlations were found with the TH of the inferior parietal lobe and middle temporal lobe. Regarding SA, Se levels exhibit positive correlations with the pars triangularis, caudal anterior cingulate, inferior parietal lobe, and banks of the superior temporal sulcus. Conversely, negative correlations were observed with the medial orbitofrontal cortex, posterior cingulate gyrus, insula, and the middle, superior, and transverse gyrus of the temporal lobe. No pleiotropy was detected.

**Results:**

This MR study indicated that Se levels causally influence the brain cortical structure.

## Graphical abstract

Using Mendelian randomization analysis and data from large‐scale genome‐wide association studies and the ENIGMA consortium, this study examined the causal relationship between selenium levels and brain cortical structure. The results revealed a correlation between selenium levels and cortical thickness, with variations observed across different brain regions. These findings provide evidence for a causal effect of selenium on brain cortical structure.

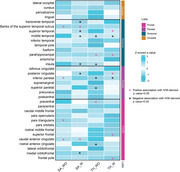

## 1 INTRODUCTION

Selenium (Se) is an essential trace element that plays a crucial role in human health through its incorporation into selenoproteins (Brown & Arthur, 2001; Kieliszek, 2019; Tinggi, 2008). Se has neuroprotective properties by counteracting oxidative stress, endoplasmic reticulum stress, and inflammation, which are believed to contribute to the development of neurodegenerative diseases. Additionally, Se promotes neuronal transmission by maintaining redox balance (Chiurchiù et al., 2016; Solovyev, 2015). The high oxygen consumption rate and sensitivity to oxidative stress of the brain highlight the importance of selenoproteins, which are closely linked to Se intake. Notably, Se has been found to influence brain function, and its deficiency or excess can have adverse effects on health. The exact safe range of Se intake remains a subject of debate. A lack of Se supply and selenoprotein dysfunction has been associated with various brain disorders, including neurodegenerative diseases (Chiurchiù et al., 2016). Se is also being explored as a potential therapeutic agent for Alzheimer's disease, multiple sclerosis, and stroke (Darvesh et al., 2010; Rajasekaran et al., 2008). Alternatively, it has been observed that elevated levels of Se, including inorganic Se compounds exceeding nutritional thresholds, can adversely affect various neurological functions and elevate the likelihood of developing type‐2 diabetes (Genchi et al., 2023).

Over the past decades, scientists traditionally believed that the brain's activity patterns, which determine our experiences, hopes, and dreams, are determined by the complex network of interconnected cells in different regions of the brain (Cozolino, 2002; Solms & Turnbull, 2018). However, a groundbreaking study from Monash University challenges the conventional understanding of brain function. Contrary to the belief that neural connections dictate brain activity, the research reveals that the overall shape of the brain plays a more significant role in shaping our thoughts, emotions, and behavior (Pang et al., 2023). Analyzing over 10,000 brain magnetic resonance imaging (MRI) scans, the study demonstrates that brain activity is strongly influenced by the geometric constraints of the brain's structure. These findings revolutionize our approach to studying brain function, offering simpler methods and potential insights into conditions like dementia and stroke. Brain shape emerges as a crucial factor in predicting brain function and understanding individual differences in behavior and neurological disorders (Pang et al., 2023). Although Se is critical for brain function, its relationship with the cortical structure of the brain is currently unknown.

Mendelian randomization (MR) is a technique that employs genetic variants as instrumental variables to investigate the causal relationship between risk factors and outcomes in disease (Burgess et al., 2015; De Ruiter et al., 2023). Two‐sample MR analysis, which utilizes summary statistics from genome‐wide association studies (GWASs) in MR studies, provides a more efficient way of analyzing large populations without individual‐level data (Cheng et al., 2020). Previous MR studies have suggested potential causal relationships between Se and various diseases, including cardiovascular disease, chronic kidney disease, prostate cancer, and schizophrenia (Deng et al., 2022; Fu et al., 2022; Rath et al., 2021; Yarmolinsky et al., 2018). However, the causal relationship between Se and cortical structure in the brain has not been fully explored. In this study, we utilized two‐sample MR analysis to explore the effects of Se on cortical structure, defined by surface area (SA) and thickness (TH), using MRI data. We conducted our analysis using publicly available GWAS data and performed subgroup analyses based on different functional regions of the brain. Our findings contribute to a better understanding of the potential relationship between Se and structural changes in the cortical regions of the brain.

## 2 METHODS

### 2.1 Selection of genetic instruments

We obtained the single‐nucleotide polymorphisms (SNPs) strongly correlated with Se levels (*p* < 5 × 10^−8^) from a GWAS meta‐analysis involving Se levels in both blood and toenail samples (Cornelis et al., 2015). The study collected toenail Se levels from 4162 individuals of European descent across four US cohorts (Chu et al., 2001; Colditz & Hankinson, 2005; Friedman et al., 1988; Jordan et al., 2007). Genetic associations were adjusted for age, sex, smoking status, and study‐specific covariates. Additionally, the study collected blood Se concentrations from 2874 pregnant women in the UK and 2603 Australian twins and their families. For these blood Se measurements, genetic associations were adjusted for age, gender, and the level of relatedness within families (Evans et al., 2013). SNPs were pruned for linkage disequilibrium (*r* ^2^ < .3) to ensure the selected instrumental variables could predict exposure independently. Then we excluded SNPs with an *F*‐statistic <10. We also checked in PhenoScanner (www.phenoscanner.medschl.cam.ac.uk), a platform with comprehensive information on the association of genotype and phenotype (Staley et al., 2016), to see whether these SNPs were associated with the potential risk factors, including body mass index, obesity, smoking, drinking, neuropsychiatric disease, hypertension, and hyperlipemia, and remove SNPs associated with any of these potential confounders at genome‐wide significance, which was similar to the previous study (Chen et al., 2021).

### 2.2 Data source for brain cortex surface area and cortex thickness

The brain cortical structure‐related data for cortical TH and SA were obtained from the ENIGMA Consortium, which is the world's largest set of brain scans (Grasby et al., 2020). The data were collected from 51,665 individuals, primarily of European descent across 60 cohorts around the world, using MRI. Meta‐analysis results of European‐ancestry participants were used in the study. The 34 regions of interest were defined based on the Desikan–Killiany atlas, and regional boundaries were determined based on the gyral anatomy labeled between the depths of the sulci (Desikan et al., 2006). The regions were averaged between both hemispheres. The study performed MR analysis to investigate the relationship between Se levels on TH and SA of the entire cortex, as well as TH and SA for 34 brain cortical regions with known functional specializations, with or without the weighted estimates of the entire brain. In total, there were 138 outcomes. The data comprising global weighted estimates indicated the SA and TH of specific regions across the SA and TH of the entire brain, whereas those without global weighted estimates indicated the SA and TH measure of specific regions, regardless of the total brain SA and TH. Table S1 provides detailed cohort information.

### 2.3 MR assumptions

To demonstrate the causal effects of MR, three fundamental assumptions must be met. The first assumption is the relevance assumption, which stipulates that the genetic instrument used should be strongly associated with the exposure under investigation (Lawlor et al., 2017; Sanderson, 2021). In our study, this assumption was satisfied as we utilized genome‐wide significant genetic variants to predict body Se concentration. The second assumption is the independence assumption, which requires that the genetic instrument used is not related to any potential confounding factors (Davies et al., 2018). Thus, we performed correlation analysis between the exposed SNPs and the outcome variables and excluded potential confounding factors related to the outcome by searching on PhenoScanner (Staley et al., 2016). The third assumption is the exclusion–restriction assumption, which states that the causal effect must be transmitted only through the exposure of interest. We employed three different methods of MR, namely, random‐effect inverse‐variance weighted (IVW), MR–Egger, and weighted median, to address the potential heterogeneity of genetic variants and pleiotropy effect and assess the validity of the exclusion–restriction assumption (Bowden et al., 2016; Kamat et al., 2019; Kanters, 2022; Yavorska & Burgess, 2017).

### 2.4 Statistical analysis

R software (version 4.1.1) with packages TwoSampleMR was used for all statistical analyses (Hemani et al., 2018).

Three different methods of MR [random‐effect IVW, MR–Egger, and weighted median] were performed to address variant heterogeneity and the pleiotropy effect (Bowden et al., 2017; Hemani et al., 2018; Verbanck et al., 2018). Leave‐one‐out (LOO) analysis and MR pleiotropy residual sum and outlier were performed to identify the outliers, with a significant influence on causal effect. IVW was used as the major outcome, whereas MR–Egger and weighted median were used to improve the IVW estimates as they could provide more robust estimates in a broader set of scenarios, despite being less efficient (wider Confidence intervals). MR–Egger allows all genetic variants to have pleiotropic effect but requires that the pleiotropic effects be independent of the variant‐exposure association. Weighted median allows for the use of invalid instruments under the assumption that at least half of the instruments used in the MR analysis are valid. In the IVW analysis, the slope of the weighted regression of the SNP‐outcome effects on the SNP‐exposure effects, where the intercept constrained to zero, represented the resulting estimate. For significant estimates, we further assessed horizontal pleiotropy using the MR–Egger intercept test and LOO analyses (He & Cui, 2021). Cochran's *Q* test was also used to identify heterogeneity. A funnel plot was used to assess the probable directional pleiotropy, like that being used to assess publication bias in meta‐analysis.

## 3 RESULTS

Eleven SNPs significantly associated with Se levels (*p* < 5 × 10^−8^) were chosen as instrumental variables (Table S2). These SNPs were independent, non‐palindromic, and considered significant. Additionally, the *F*‐statistics for all the genetic instruments were greater than 10, indicating strong strength. Furthermore, these instrumental variables are mainly situated on chromosomes 5 and 21, with several SNPs clustered in proximity to the DMGDH, CBS, and BHMT genes. Among the instrumental variables we identified, no SNP showed a significant association with cortical structure. Meanwhile, we tested whether the significant estimate was violated by risk factors and conducted an SNP lookup in the PhenoScanner. We found no association between these SNPs and the potential risk factors, including body mass index, obesity, smoking, drinking, neuropsychiatric disease, hypertension, and hyperlipemia.

**FIGURE 1:**
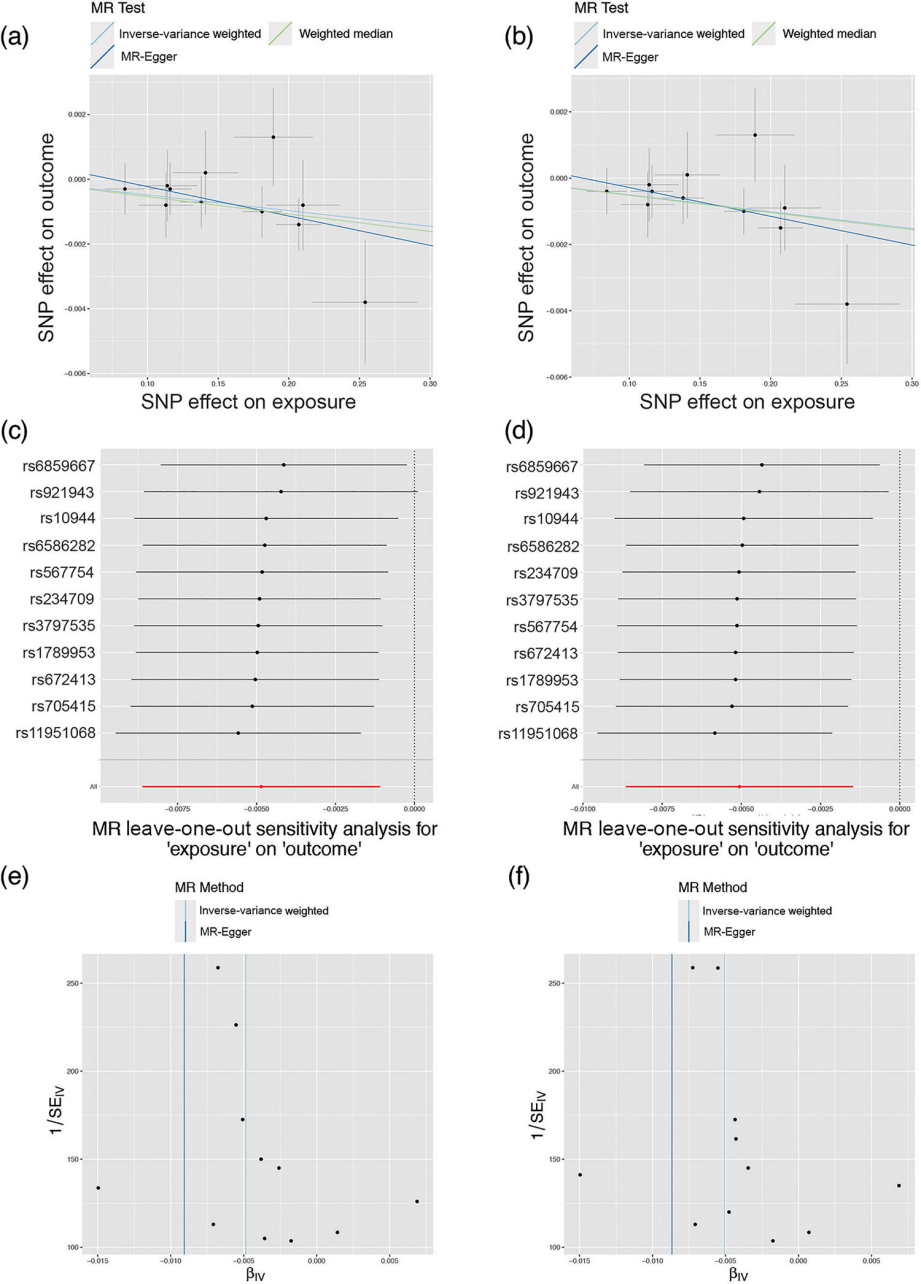
(a and b) Scatter plots show the significant estimates from genetically predicted selenium levels on full cortical thickness with or without global weighted. Leave‐one‐out plots (c and d) display the significant estimates from genetically predicted selenium levels on full cortical thickness with or without global weighted. Funnel plots (e and f) illustrate the significant estimates from genetically predicted selenium levels on full cortical thickness with or without global weighted.

We conducted a comprehensive MR analysis to investigate the relationship between genetically predicted Se levels and both global SA/TH and 34 functional gyri, with and without global weighting. Our findings revealed the presence of several notable functional gyri that may be potentially influenced by Se levels. At the global level, blood Se levels were found to decrease TH (*β*_TH_ = −0.00485 mm, SE_TH_ = 0.00192, *p* = .0115, Figure 1a) but had no causal relationship with SA (*β*_SA_ = 400.34 mm^2^, SE_SA_ = 277.65, *p* = .149) with global weighted. Similar finding was in without global weighted, blood Se levels were found to decrease TH (*β*_TH_ = −0.00506 mm, SE_TH_ = 0.00183, *p* = .00570, Figure 1a) but had no causal relationship with SA (*β*_SA_ = 389.92 mm^2^, SE_SA_ = 266.94, *p* = .144). The *p* value for MR–Egger intercept is >.05. No outliers were identified with the LOO plot and funnel plots (Figure 1c–f).

**FIGURE 2:**
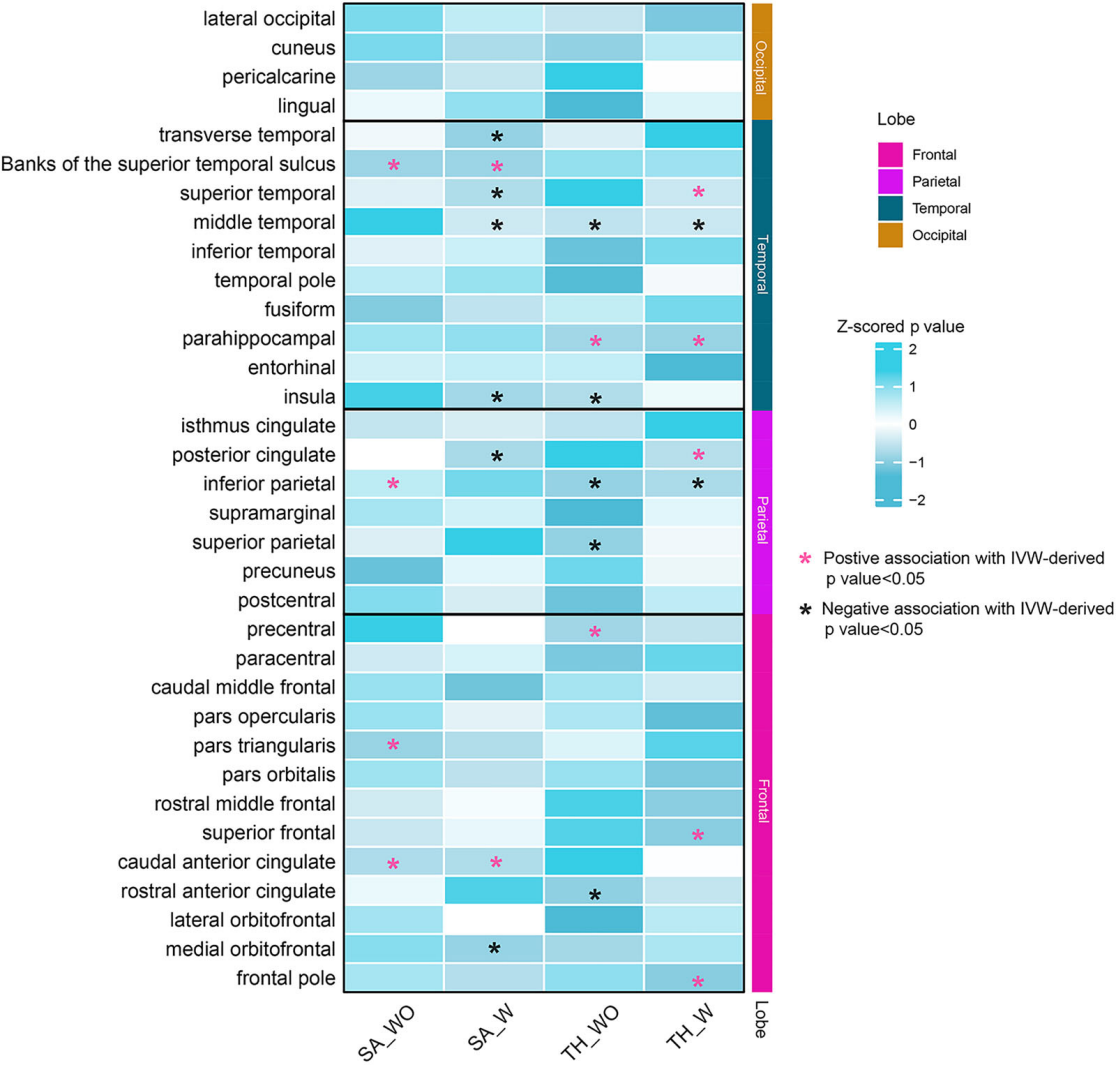
Inverse‐variance weighted (IVW) estimates of selenium levels on brain cortical structure as defined using magnetic resonance imaging‐measured brain cortical surface area and thickness. The color of each block represents the IVW‐derived *p* values of every Mendelian randomization (MR) analysis. (SA_WO, surface area without global weighted, SA_W, surface area with global weighted, TH_WO, thickness without global weighted, TH_W, thickness with global weighted, *p < .05).

Table 1 illustrates the significant estimates between Se levels and the TH and SA of different brain regions. Overall, the Se level is closely related to TH and SA in some brain lobes and influenced by global_weighted correction (Figure 2). For TH, Se levels demonstrate inverse correlations with the middle temporal and inferior parietal, while exhibiting positive correlations with the parahippocampal gyrus regardless of global weighted correction. Regarding SA, Se levels exhibit positive correlations with the dimensions of the pars triangularis, caudal anterior cingulate, inferior parietal region, and banks of the superior temporal sulcus. Conversely, negative correlations are observed with the medial orbitofrontal region, posterior cingulate, insula, middle, superior, and transverse temporal gyri.

**TABLE 1:** Significant Mendelian randomization estimates from selenium levels on genetically predicted cortical structure with or without global weighted.

Exposure	Outcome		Outcome	IVW‐derived *p* value	*β*	SE	Cochran's *Q*–derived *p* value	MR–Egger intercept–derived *p* value
**Selenium**	**With global weighted**	**Surface area**	bankssts	.039	4.448	2.156	.730	.500
			Caudal anterior cingulate	.008	5.070	1.918	.989	.180
			Insula	.009	−8.562	3.275	.507	.655
			Medial orbitofrontal	.032	−5.624	2.622	.629	.141
			Middle temporal	.036	−10.878	5.174	.220	.719
			Posterior cingulate	.011	−5.596	2.204	.623	.432
			Superior temporal	.002	−15.134	4.896	.384	.211
			Transverse temporal	.016	−2.363	.980	.834	.608
		**Thickness**	Frontal pole	.023	.011	.005	.363	.187
			Inferior parietal	.007	−.004	.002	.272	.668
			Middle temporal	.030	−.006	.003	.250	.091
			Parahippocampal	.004	.015	.005	.515	.481
			Posterior cingulate	.033	.005	.002	.832	.862
			Superior frontal	.028	.005	.002	.330	.026
			Superior temporal	.045	.005	.003	.359	.076
	**Without global weighted**	**Thickness**	Inferior parietal	.002	−.008	.003	.638	.485
			Precentral	.003	−.008	.003	.817	.962
			Middle temporal	.010	−.009	.003	.546	.064
			Insula	.019	−.007	.003	.971	.155
			Superior parietal	.021	−.006	.003	.392	.969
			Parahippocampal	.031	.012	.006	.575	.527
			Rostral anterior cingulate	.042	−.008	.004	.958	.872
		**Surface area**	Caudal anterior cingulate	.006	6.416	2.352	.980	.141
			Pars triangularis	.025	8.234	3.677	.564	.471
			bankssts	.041	5.430	2.661	.883	.299
			Inferior parietal	.043	23.969	11.855	.889	.155

Abbreviations: banks of the superior temporal sulcus, bankssts; IVW, inverse‐variance weighted.

For all estimates, Cochran's *Q* test, MR–Egger intercept test, LOO analyses, and funnel plot were used to assess horizontal pleiotropy. All *p* values of MR–Egger intercept tests were >.05 except for estimates of Se on TH of superior frontal area with global weighted, indicating that no horizontal pleiotropy existed. Scatter plots, LOO analyses, and funnel plots of significant estimates are shown in Figures S1–S3. The estimates were not biased by SNP, indicating that estimates were not violated. Cochran's *Q*–derived *p* values were all >.05.

## 4 DISCUSSION

Se exerts its various biological functions primarily through selenoproteins that contain selenocysteine in their active centers (Steinbrenner & Sies, 2009). Se has been identified as playing a role in several neurodegenerative disorders, including Alzheimer's and Parkinson's disease, as well as being closely associated with functions, such as motor performance, coordination, memory, and cognition (Solovyev et al., 2018; Wrobel et al., 2016). Their functions range from well‐established antioxidant activities to less understood mechanisms that modulate mitochondrial function and response to brain pathology (Cardoso et al., 2015). Although numerous studies have assessed the association between Se levels and brain function and diseases, the relationship between Se levels and brain structure remains largely unknown. This article represents the first analysis using MR to explore the causal relationship between blood and toenail Se levels and the cortical structure of the brain. Our findings suggest that Se levels in the blood may influence the structure of the cortical brain, revealing the interplay between Se levels and brain structure, as well as potential functions.

It is worth noting that the identified instrument SNPs in this study are located on chromosomes 5 and 21, near the DMGDH, CBS, and BHMT genes. Previous GWAS of blood Se by Evans et al. (2013) and GWAS of toenail Se by Cornelis et al. (2015) both reported significant SNPs mapping to the DMGDH and BHMT regions, which are closely associated with increased Se concentrations following Se supplementation. Both the dimethylglycine dehydrogenase encoded by DMGDH and the betaine‐homocysteine *S*‐methyltransferase encoded by BHMT have been demonstrated to be implicated in homocysteine (Hcy) metabolism, suggesting a possible link between Se exposure and the Hcy metabolic pathway (Maclean et al., 2019). Additionally, CBS, which encodes cystathionine β‐synthase, exhibits a close correlation with elevated levels of Se and Hcy (Van Meurs et al., 2013). CBS functions as a regulatory component within the *trans*‐sulfuration pathway. Polymorphisms in CBS can lead to impaired clearance of Hcy, subsequently affecting the synthesis of Met cyle and *S*‐adenosylmethionine (SAM), as well as the excretion of Se (Fairweather‐Tait et al., 2011; Jackson et al., 2013).

Interestingly, our study revealed a negative correlation between Se levels and the TH of the cerebral cortex, regardless of global weighting. This finding deviates from our initial expectations. The cortical structure of the brain, particularly cortical TH, serves as an imaging marker indicating cognitive decline and human intelligence (Sørensen et al., 2017). In a study involving 182 healthy individuals aged 9–24, a comprehensive positive association between cortical TH and overall intelligence was observed, with the association being influenced by the age of the participants (Menary et al., 2013). Specifically, cortical TH showed a positive correlation with intelligence in older individuals but a negative correlation in younger individuals. Besides, previous research has demonstrated a positive relationship between Se status in older adults and cognitive performance, suggesting that optimizing Se intake could help mitigate the risk of age‐related cognitive decline (Ferry & Roussel, 2011). However, a cross‐sectional study involving 154 older adults conducted multiple linear regression analyses, which failed to reveal any association between plasma Se, cognitive performance, inflammatory markers, or neurotrophic factors (Cardoso et al., 2018). These findings could be attributed to the high serum Se status in the study population. Furthermore, Ma et al. identified that elevated levels of potentially toxic inorganic forms of Se in the cerebrospinal fluid could predict the transition from mild cognitive impairment to Alzheimer's dementia (Vinceti et al., 2017). Therefore, it should be noted that the effects of Se can manifest as both beneficial and deleterious, contingent upon the dosage and form (morphology) of Se, as well as the individual's age. Serum Se levels exhibit a biphasic dose response characterized by beneficial effects at low doses and inhibitory or toxic effects at high doses.

Simultaneously, it is essential to consider specific brain lobes and their respective functions when examining the relationship between Se levels and brain structure. Our analysis unveiled a positive correlation between Se levels and the TH of lobes associated with executive cognitive functions, such as the parahippocampal gyrus, superior frontal gyrus, frontal pole, and posterior cingulate gyrus. This finding aligns with the research conducted by Leiter et al. (2022), who demonstrated that Se plays a mediating role in exercise‐induced adult neurogenesis and mitigates learning deficits caused by hippocampal injury and aging. Notably, exercise enhances the systemic levels of selenoprotein P1, facilitating the transport of Se from the bloodstream to the dentate gyrus in the hippocampus. Consequently, neural progenitor cell proliferation increases, significantly restoring structural and functional impairments in the aging hippocampus. Moreover, irrespective of global weighting, we observed a negative correlation between Se levels and the TH of the inferior parietal lobe and middle temporal gyrus. These brain regions are known for their involvement in spatial attention, multimodal sensory integration, oculomotor control, language comprehension, and auditory processing. However, further investigation is necessary to fully understand the relationship between cortical TH in these areas and their corresponding functions to elucidate the underlying mechanisms.

Our study did not find a significant association between Se levels and the overall cortical SA. The cortical SA is affected by genetic variation, which can modify the gene regulatory activity of neural progenitor cells during embryonic development (Corbin et al., 2008). On the other hand, cortical TH is affected by active regulatory elements and reflects post‐mid‐fetal development processes, such as myelination, branching, or pruning (Tau & Peterson, 2010). Interestingly, there is a negative genetic correlation between cortical SA and average TH, where genetic changes in a specific subregion result in increased SA but decreased TH (Grasby et al., 2020; Rimol et al., 2010). Similarly, we observed a negative correlation between Se levels and cortical SA and a positive correlation with cortical TH in the posterior cingulate and superior temporal gyrus. These findings provide valuable insights into the role of Se in the structural changes occurring in these cortical regions.

Besides, we did uncover a negative correlation between Se levels and the SA with several gyrus of the temporal lobe. Up to now, a decrease in the SA of the temporal lobe has been strongly associated with a decline in cognitive function, the onset of Alzheimer's disease, and the occurrence of epilepsy (Dickerson et al., 2009; Taylor et al., 2015). Our observation may contradict the potential therapeutic role of Se supplementation in managing epilepsy and AD (Casillas‐Espinosa et al., 2023; Varikasuvu et al., 2019). However, Vinceti et al. (2022) found that patients with mild cognitive impairment exhibit elevated concentrations of inorganic Se in their cerebrospinal fluid, and when selenate concentrations exceed 0.2 ng/mL, they are negatively correlated with hippocampal volume. Therefore, it reminds us of the potential adverse consequences when considering Se supplementation.

Several limitations should be considered when interpreting our findings. First, although our genetic analysis focused on individuals of European ancestry, it is crucial to emphasize that Se status exhibits significant heterogeneity between American and European populations, owing to geographical variances and diverse soil compositions (Cardoso et al., 2015). Consequently, the generalizability of our findings to other ethnic groups remains uncertain. Furthermore, the data limitations of individual‐level clinical data and precise Se measurements posed challenges in conducting subgroup analyses and identifying the specific range of Se levels that influence cortical structural changes, thus restricting the clinical applicability of our findings. Third, the underlying biological mechanisms that connect Se to brain structure changes need further research to elucidate the biological rationale. Lastly, it is important to consider the potential influence of sample overlap on our results.

## CONCLUSION

This study represents the first comprehensive MR analysis to elucidate the relationship between Se levels and cortical brain structure. It provides genetic evidence and novel insights for researchers exploring the association between Se levels and cortical brain structure, particularly in different functional brain regions. However, further investigation is needed to uncover the biological mechanisms underlying the relationship between Se and structural changes in the brain.

## AUTHOR CONTRIBUTIONS

**Xiaowei Zhang**: Conception; data curation; validation; visualization; and drafting and revising the work. **Yuqing Zhong**: Data curation; validation; visualization. **Kejun He**: Data curation; validation; visualization.

## CONFLICT OF INTEREST STATEMENT

The authors state that there are no conflicts of interest in relation to this study.

## INSTITUTIONAL REVIEW BOARD STATEMENT

This study utilized publicly available databases and therefore does not require ethical approval.

## INFORMED CONSENT STATEMENT

As the study is based on a secondary analysis of existing publicly available datasets, informed consent is not applicable.

### PEER REVIEW

The peer review history for this article is available at https://publons.com/publon/10.1002/brb3.3296.

## Supporting information

Supplementary Figure Information

Table_S1 Descriptions of study cohorts participating in Grasby's study.
Table_S2 Eleven instrumental SNPs represent genetically predicted selenium levels.

## Data Availability

The datasets analyzed in this study are publicly available and can be accessed at the following links: https://pubmed.ncbi.nlm.nih.gov/25343990/, https://enigma.ini.usc.edu/.
