# Supplementary material for: The causal effects between selenium levels and the brain cortical structure: A two‐sample Mendelian randomization study
Source: Brain Behav. 2023 Oct 30;13(12):e3296. doi: 10.1002/brb3.3296 (PMC10726828; doi:10.1002/brb3.3296)
Supplement: Supplementary file 1 — Supplementary Figure Information [file BRB3-13-e3296-s002.docx]

Supplementary Information

**The causal effects between selenium levels and the brain cortical structure: A two-sample mendelian randomization study.**

**Xiaowei Zhang, Yuqing Zhong, Kejun He.**

**Figure S1.** Scatter plots of significant estimates from genetically predicted selenium levels on with global weighted SA of the (a) bankssts (b) caudal anterior cingulate (c)insula (d) medial orbitofrontal (e) middle temporal (f) posterior cingulate (g) superior temporal (h) transverse temporal, respectively; With global weighted TH of the (i) frontal pole, (j) inferior parietal, (k) middle temporal, (l) parahippocampal, (m) posterior cingulate, (n) superior frontal, and (o) superior temporal, respectively; Without global weighted SA of the (p) caudal anterior cingulate, (q) inferior parietal, (r) pars triangularis, and (s) bankssts, respectively; Without global weighted TH of the (t) inferior parietal, (u) precentral, (v) middle temporal, (w) insula, (x) superior parietal, (y) parahippocampal, and (z) rostral anterior cingulate.

**Figure S2.** Leave-one-out plots of significant estimates from genetically predicted selenium levels on with global weighted SA of the (a) bankssts (b) caudal anterior cingulate (c)insula (d) medial orbitofrontal (e) middle temporal (f) posterior cingulate (g) superior temporal (h) transverse temporal, respectively; With global weighted TH of the (i) frontal pole, (j) inferior parietal, (k) middle temporal, (l) parahippocampal, (m) posterior cingulate, (n) superior frontal, and (o) superior temporal, respectively; Without global weighted SA of the (p) caudal anterior cingulate, (q) inferior parietal, (r) pars triangularis, and (s) bankssts, respectively; Without global weighted TH of the (t) inferior parietal, (u) precentral, (v) middle temporal,(w) insula, (x) superior parietal, (y) parahippocampal, and (z) rostral anterior cingulate.

**Figure S3.** Funnel plots of significant estimates from genetically predicted selenium levels on with global weighted SA of the (a) bankssts (b) caudal anterior cingulate (c)insula (d) medial orbitofrontal (e) middle temporal (f) posterior cingulate (g) superior temporal (h) transverse temporal, respectively; With global weighted TH of the (i) frontal pole, (j) inferior parietal, (k) middle temporal, (l) parahippocampal, (m) posterior cingulate, (n) superior frontal, and (o) superior temporal, respectively; Without global weighted SA of the (p) caudal anterior cingulate, (q) inferior parietal, (r) pars triangularis, and (s) bankssts, respectively; Without global weighted TH of the (t) inferior parietal, (u) precentral, (v) middle temporal, (w) insula, (x) superior parietal, (y) parahippocampal, and (z) rostral anterior cingulate.
