# Supplementary material for: The causal effects between selenium levels and the brain cortical structure: A two‐sample Mendelian randomization study
Source: Brain Behav. 2023 Oct 30;13(12):e3296. doi: 10.1002/brb3.3296 (PMC10726828; doi:10.1002/brb3.3296)
Supplement: Supplementary file 2 — Table_S1 Descriptions of study cohorts participating in Grasby's study. Table_S2 Eleven instrumental SNPs represent genetically predicted selenium levels. [file BRB3-13-e3296-s001.docx]

Table S1. Descriptions of study cohorts participating in Grasby’s study.

| **Cohort** | **Ancestry** | **Study Design** | **Total N** | **Females** | **Mean Age** | **SD** | **Min Age** | **Max Age** | **Healthy N** |
| --- | --- | --- | --- | --- | --- | --- | --- | --- | --- |
| 1000BRAINS | European | Population-based | 775 | 346 | 67.3 | 6.7 | 53.4 | 85.4 | 775 |
| ADNI1 | European | Case-control (AD, MCI, healthy control) | 735 | 299 | 74.8 | 6.8 | 54.0 | 90.0 | 204 |
| ADNI2GO | European | Case-control (AD, MCI, healthy control) | 649 | 297 | 72.4 | 7.1 | 55.0 | 91.4 | 564 |
| ALSPAC^a^ | European | Population-based | 391 | 0 | 19.6 | 0.9 | 18.0 | 21.5 | 391 |
| ASRB | European | case-control | 233 | 138 | 38.5 | 11.3 | 19.0 | 64.0 | 75 |
| BETULA | European | Population-based | 311 | 169 | 62.4 | 13.3 | 25.5 | 81.3 | 311 |
| BIG-Affy | European | Population-based | 1180 | 688 | 22.6 | 3.8 | 18.0 | 40.0 | 1180 |
| BIG-PsychChip | European | Population-based | 432 | 206 | 22.5 | 4.4 | 17.0 | 44.0 | 432 |
| BONN | European | Population-based | 102 | 0 | 38.2 | 6.6 | 29.0 | 50.0 | 102 |
| BrainScale | European | Population-based Twin Study | 242 | 131 | 10.0 | 1.3 | 9.0 | 15.0 | 242 |
| CARDIFF | European | Population-based | 270 | 194 | 24.8 | 6.9 | 18.0 | 58.0 | 270 |
| DNS-V3 | European | Population-based | 324 | 168 | 19.7 | 1.2 | 18.0 | 22.0 | 324 |
| DNS-V4 | European | Population-based | 191 | 108 | 19.9 | 1.2 | 18.0 | 22.0 | 191 |
| EPIGEN | European | Epilepsy cases | 178 | 104 | 38.4 | 13.2 | 14.0 | 85.0 | 0 |
| FOR2107 | European | Population-based plus Affective disorders cases | 785 | 474 | 34.4 | 13.0 | 18.0 | 65.0 | 416 |
| GIG | European | Population-based | 283 | 168 | 24.2 | 2.4 | 19.0 | 31.0 | 283 |
| GSP | European | Population-based | 442 | 251 | 21.4 | 3.2 | 18.0 | 35.0 | 442 |
| HUBIN | European | Case-control (SCZ, healthy controls) | 177 | 55 | 41.9 | 8.2 | 19.4 | 56.3 | 97 |
| HUNT | European | Population-based | 876 | 462 | 58.9 | 4.2 | 50.5 | 66.8 | 876 |
| IMAGEN | European | Population-based | 1358 | 725 | 14.6 | 0.4 | 12.9 | 17.2 | 1358 |
| IMpACT | European | Case-control (ADHD, healthy controls) | 238 | 140 | 40.8 | 12.0 | 20.0 | 70.0 | 113 |
| LBC1936 | European | Population-based | 604 | 285 | 72.7 | 0.7 | 71.0 | 74.2 | 604 |
| LIBD | European | Case-control (SCZ, healthy controls, unaffected siblings) | 484 | 214 | 33.2 | 10.1 | 18.6 | 61.6 | 310 |
| MCIC | European | Case-control (SCZ, healthy controls) | 162 | 56 | 33.7 | 11.2 | 18.0 | 59.0 | 94 |
| MooDS | European | Population-based | 282 | 129 | 33.6 | 9.8 | 18.0 | 51.0 | 282 |
| MPIP | European | Case-control (MDD, healthy controls) | 550 | 318 | 48.3 | 13.3 | 18.0 | 87.0 | 177 |
| MPRC | European | Case-control (SCZ, healthy controls) | 387 | 205 | 37.2 | 14.6 | 10.0 | 79.0 | 214 |
| MÜNSTER | European | Case-control (MDD, healthy controls) | 985 | 561 | 35.8 | 12.1 | 17.0 | 65.0 | 741 |
| NCNG | European | Population-based | 321 | 218 | 51.6 | 16.7 | 19.4 | 82.3 | 321 |
| NESDA | European | Case-control (depression, anxiety, healthy controls) | 254 | 171 | 37.5 | 10.2 | 18.0 | 57.0 | 55 |
| NeuroIMAGE | European | Case-control (ADHD, healthy controls) | 210 | 68 | 17.1 | 3.2 | 8.2 | 25.0 | 61 |
| NTR | European | Population-based Twin Study | 322 | 197 | 29.4 | 11.0 | 12.0 | 56.0 | 322 |
| OATS | European | Population-based Twin Study | 360 | 237 | 70.5 | 5.1 | 65.0 | 89.0 | 360 |
| PAFIP | European | Case-control (SCZ, healthy controls) | 112 | 42 | 28.3 | 8.0 | 16.1 | 50.7 | 14 |
| PDNZ | European | Case-control (Parkinson's disease, healthy controls) | 164 | 56 | 68.2 | 7.8 | 45.5 | 81.9 | 47 |
| PING | European | Population-based | 337 | 151 | 11.8 | 4.7 | 3.4 | 20.8 | 337 |
| PPMI | European | Case-control | 414 | 137 | 61.7 | 9.6 | 30.6 | 84.9 | 124 |
| QTIM | European | Population-based Twin Study | 996 | 645 | 22.4 | 3.3 | 15.4 | 30.1 | 996 |
| SHIP | European | Population-based | 1118 | 579 | 55.8 | 12.8 | 30 | 90 | 1118 |
| SHIP-Trend | European | Population-based | 891 | 499 | 50.4 | 13.5 | 22 | 81 | 891 |
| Sydney MAS | European | Population-based | 494 | 274 | 78.4 | 4.7 | 70.5 | 90.1 | 494 |
| SYS | European | Family-based study | 1675 | 884 | 28.3 | 17.4 | 11 | 65.4 | 1675 |
| TCD-NUIG | European | Case-control (healthy control, schizophrenia) | 192 | 108 | 29.9 | 10.4 | 18 | 63 | 156 |
| TOP | European | Case-control (SCZ, BD, other psychoses, healthy controls) | 505 | 253 | 35.2 | 10.2 | 18.2 | 64.8 | 216 |
| TOP3T | European | Case-control (SCZ, BD, other psychoses, healthy controls) | 400 | 181 | 33.2 | 11.9 | 18 | 78 | 296 |
| UiO2016 | European | Case-control | 229 | 98 | 31.8 | 10.0 | 15.6 | 60.5 | 90 |
| UiO2017 | European | Case-control | 308 | 173 | 42.1 | 17.9 | 13.1 | 89.0 | 247 |
| UKBB | European | Population-based | 10083 | 5261 | 62.8 | 7.4 | 46.4 | 46.4 | 10083 |
| UMCU | European | Case-control / family study (SCZ, BD, offspring, healthy controls) | 698 | 373 | 33.1 | 14.1 | 9 | 67 | 548 |

Table S2. 11 instrumental SNPs represented genetically predicted selenium levels.

| **SNP** | **Nearby gene** | **effect_allele** | **other_allele** | **EAF** | **Beta** | **SE** | ***P* value** |
| --- | --- | --- | --- | --- | --- | --- | --- |
| rs672413 | ARSB | A | G | 0.32 | 0.116 | 0.015 | 1.05E-14 |
| rs705415 | DMGDH | C | T | 0.86 | 0.141 | 0.023 | 8.76E-10 |
| rs3797535 | DMGDH | T | C | 0.08 | 0.21 | 0.026 | 6.64E-16 |
| rs11951068 | DMGDH | A | G | 0.07 | 0.189 | 0.028 | 1.48E-11 |
| rs921943 | DMGDH | T | C | 0.29 | 0.207 | 0.016 | 2.76E-38 |
| rs10944 | BHMT2 | T | G | 0.49 | 0.181 | 0.014 | 3.11E-38 |
| rs567754 | BHMT | C | T | 0.66 | 0.138 | 0.015 | 3.58E-20 |
| rs6859667 | HOMER1 | C | T | 0.04 | 0.254 | 0.037 | 6.66E-12 |
| rs6586282 | CBS | C | T | 0.83 | 0.113 | 0.019 | 2.72E-09 |
| rs1789953 | CBS | T | C | 0.14 | 0.114 | 0.021 | 5.68E-08 |
| rs234709 | CBS | C | T | 0.55 | 0.084 | 0.014 | 1.97E-09 |
